# Measuring differential gene expression by short read sequencing: quantitative comparison to 2-channel gene expression microarrays

**DOI:** 10.1186/1471-2164-10-221

**Published:** 2009-05-12

**Authors:** Joshua S Bloom, Zia Khan, Leonid Kruglyak, Mona Singh, Amy A Caudy

**Affiliations:** 1 Department of Molecular Biology, Princeton University, New Jersey, USA; 2 Department of Computer Science, Princeton University, New Jersey, USA; 3 Lewis-Sigler Institute of Integrative Genomics, Princeton University, New Jersey, USA; 4 Department of Ecology and Evolutionary Biology, Princeton University, New Jersey, USA; 5 Howard Hughes Medical Institute, Princeton University, New Jersey, USA

## Abstract

**Background:**

High-throughput cDNA synthesis and sequencing of poly(A)-enriched RNA is rapidly emerging as a technology competing to replace microarrays as a quantitative platform for measuring gene expression.

**Results:**

Consequently, we compared full length cDNA sequencing to 2-channel gene expression microarrays in the context of measuring differential gene expression. Because of its comparable cost to a gene expression microarray, our study focused on the data obtainable from a single lane of an Illumina 1 G sequencer. We compared sequencing data to a highly replicated microarray experiment profiling two divergent strains of *S. cerevisiae*.

**Conclusion:**

Using a large number of quantitative PCR (qPCR) assays, more than previous studies, we found that neither technology is decisively better at measuring differential gene expression. Further, we report sequencing results from a diploid hybrid of two strains of *S. cerevisiae *that indicate full length cDNA sequencing can discover heterozygosity and measure quantitative allele-specific expression simultaneously.

## Background

RNA sequencing has been one of the first applications of the revolutionary ultrahighthroughput DNA sequencing technologies [1-9]. This work has demonstrated the power of deep RNA sequencing for determining comparative gene expression and for discovering the full extent of 5' and 3' untranslated regions, novel splice junctions, novel transcripts, alternate transcription start sites, and rare transcripts. In this work, we explore the quantitative power of Illumina sequencing-by-synthesis, analyze the same samples using DNA microarrays, and extensively validate our results using quantitative PCR. Because one of the powerful advantages of new sequencing technologies is the ability to characterize sequence variation, we also determined the accuracy of RNA sequencing for discovering sequence variation.

In this study, we examined the use of full length cDNA sequencing for measuring differential gene expression in two divergent haploid strains of *S. cerevisiae *[10]. Because of the comparable cost of a single lane of an Illumina 1 G sequencer and a gene expression microarray, we compared the data obtained from one lane of the Illumina 1 G to a highly replicated collection of 2-channel Agilent array data from a study that profiled two divergent strains of *S cerevisiae*: BY4716 and RM11-1a[11]. In the present study we sequenced cDNA prepared from the same mRNA samples used in the microarray study, controlling for both biological variance and variance in mRNA isolation simultaneously.

In contrast to gene expression microarrays, which measure gene expression by quantifying the hybridized and labeled cDNA, the Illumina 1 G measures gene expression by sequencing fragments of cDNA. The sequencing reads are aligned to a known reference genome sequence, and quantitative gene expression values are obtained by counting the number of these fragments matching a known open reading frame (ORF). One of the consequences of the adapter ligation cloning approach used in this study is that strand origin of the expressed transcript is lost. If two genes overlap, one on the Watson and the other on the Crick strand, their expression levels will be indistinguishable for the region of overlap.

The primary limits of the Illumina 1 G sequencer are short read length and total number of reads obtainable in a single lane of a sequencing reaction. Read length at the time of our study was 32 base pairs, and the total number of reads obtainable in a sequencing reaction was about 5 million. Short read length increases the likelihood of reads that map to multiple locations. If these multiple-mapping reads are not removed, they will contribute mixed signal that does not distinguish expression of different genes. The second limitation, the number of reads, is more problematic given that a small subset of transcripts are highly abundant (e.g. noncoding RNAs such as the ribosomal structural RNA). These highly abundant transcripts will be sequenced more frequently, leaving few reads for transcripts that are in low abundance. As a result, the low abundance transcripts will be more affected by sampling error. Microarray analysis is much less limited by the presence of highly abundant transcripts, because each mRNA binds independently to its complementary sequence feature on a microarray.

In addition to quantitative measurement of gene expression, we report initial results showing that full length cDNA sequencing can discover heterozygosity and measure allele specific expression (ASE) in diploid strains of yeast. Many organisms are primarily found in nature as highly heterozygous, diploid individuals. Further, many species, including some major crop plants, are descended from multiple hybridization events and are highly polyploid [12]. The large, closely related gene families in such species can complicate array analysis but in most cases are distinguishable by sequencing analysis. Finally, discovering the genome-wide extent of heterozygosity in any diploid species with a large genome can be impractical by full genome re-sequencing, even with the aid of high throughput sequencing methods. By examining base pairs exlusively within coding regions, we find it is possible to identify heterozygous sites as well as their relative, or allele-specific, expression.

## Results

### Illumina Read Alignment

The basis of our comparison was a DNA microarray array experiment in which the gene expression in two different haploid strains of *S. cerevisiae*, RM11-1a and BY4716, in two different growth media was measured using six biological replicates (GEO accession number GSE9376) [11]. Two color Agilent arrays were used, with a common reference consisting of a mixture of all of the original conditions and strains. Using the same RNA samples profiled in the array replicates, we sequenced full length cDNA corresponding to each strain and condition with a single lane of the Illumina 1 G sequencer.

Differential gene expression information from the Illumina 1 G sequencer was obtained by aligning reads to a reference genome and counting the number of reads that overlap each ORF. Samples were denoted BYg, BYe, RMg, RMe where BY indicates strain BY4716 and RM indicates RM11-1a. The lower-case e and g denote two nutrient conditions: ethanol and glucose as carbon sources. We used a simple alignment strategy that assumed a low error rate and determined the alignment with the entire reference genome with the fewest number of differences (insertions, deletions, and substitutions) of the reads. Between 91.6% and 96.2% of the reads obtained from a single lane of the Illumina 1 G sequencer aligned with the reference *S. cerevisiae *sequence [see Additional file 1, Table S1]. The BY4716 strain has few differences from the S288C reference strain. Using the unfinished assembly of the RM11-1a genome sequence from the Broad Institute, we found 5974 genes that were reciprocal best matches with at least 98% sequence identity between the two strains across alignments of ORFs, including 1 kb upstream and 1 kb downstream sequence[13]. Expression values were only computed for these 5974 genes using the Illumina 1 G data.

We separated reads into unique reads that aligned to a single location in the genome and reads that aligned to multiple locations. Among the reads that mapped to a unique position, we separated reads into those that mapped to an ORF, a ribosomal RNA (rRNA) gene, or a transfer RNA (tRNA) gene. We observed that the vast majority of the unique reads originated from ORFs [see Additional file 1, Table S2]. The distribution among multiple-mapping reads was different (see Table S3). Among these reads, most originated from rRNA genes. Even though we reduced the population of rRNA in our samples by depleting ribosomal small and large subunit RNAs with a LNA probe and by using reverse transcription with an oligo-dT primer, between 30.9%–60.4% of the total number of reads originated from rRNAs [see Additional file 1, Table S3]. Furthermore, one of the consequences of the short read length of the Illumina 1 G is that reads from rRNAs might also align to ORFs of protein-coding genes. In the five samples, we found that between 4.6%–10.4% of the total number of reads matched both an rRNA gene and an ORF. A smaller fraction of reads, 1.9%–3.3%, aligned to two or more ORFs. In all further analyses we used only reads that uniquely align to an ORF, including the few reads that mapped to more than one location within a single ORF.

### Statistically Significant Differential Gene Expression

**Figure 1:**
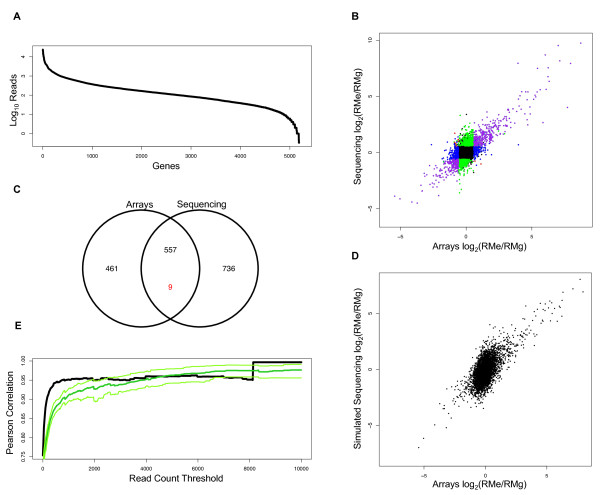
**Sequencing and arrays show correlated differential expression but sequencing is more susceptible to sampling error**. Read counts are not evenly distributed across genes. For the RMg sample, log_10 _read counts per gene are shown (A), with genes ordered by abundance. The log_2 _ratio of the medians of six replicate microarray experiments for RM in ethanol vs RM in glucose is compared to the log_2 _ratio of sequencing read counts. The methods are correlated (R = 0.75356, 95% CI: 0.7236–0.785). Colors indicate significantly differentially expressed genes at a FDR<1% and 1.5 fold or greater change, where significance is determined using Fisher's exact test for the sequencing data and the Mann-Whitney test for the array data. Purple indicates significantly different by both methods, green is significantly different by sequencing only, blue is significantly different by microarrays only, and red is significant by both methods but with opposite directionality (B). Data from (B) but represented as a Venn diagram of significant differences; note in red the 9 genes measured as significantly changed but in opposite directions (C). The results from (B) can be modeled by sampling from binomial distributions for each gene. Here a single random sampling is shown (D). The correlation of log_2 _expression ratios determined by microarrays and sequencing is highly dependent on the number of read counts per gene. For both the actual data (black), and simulated data (green) with 95% confidence intervals (light green), correlation improves as the thresholds for sequence coverage increase (E).

To carefully assess the ability of the sequencer to detect differential expression we focused our analysis on the RMe and RMg samples, which show significant differential expression between the two environmental conditions, had similar data quality, and contain differences from the reference sequence. Analysis of the other samples is summarized in [see Additional file 1, Table S4]. We obtained 1,919,687 reads for RMe and 2,719,827 reads for RMg that aligned to ORFs and passed our filtering criteria, resulting in 5186 genes covered by at least one read. For each ORF a digital measurement of expression was obtained by counting the total number of reads that align to it (Fig. 1A). Differential gene expression between RMe and RMg was determined by taking the log_2 _ratio of RMe to RMg counts, normalizing for the different total number of read counts between the two samples. For the 4697 genes that passed quality criteria for both the microarrays and sequencer, the resulting ratio was highly correlated (R = 0.75356, bootstrap 95% CI: 0.7236–0.7851) between microarrays and the sequencing data (Fig. 1B).

We identified the subset of differentially expressed genes for each platform. We detected significant differential expression for 1027 genes by arrays and 1303 genes by sequencing, with 566 genes detected as differentially expressed by both platforms (Fig. 1C). The subsets of genes detected as differentially expressed in a single technology had distinct array intensities and read coverage. The subset of genes that were called significantly differentially expressed only by arrays had significantly lower read counts in the sequencing data than the set of all genes examined (t = -5.2306, df = 1132.873, p < 2.012 × 10^-7^) as well as significantly lower average array intensity (t = -5.3667, df = 1137.305, p < 9.175 × 10^-8^), suggesting that differential expression of genes with low expression levels is detected more readily by arrays. The subset of genes that were called significantly differentially expressed by sequencing alone had higher read counts than the set of all genes examined (t = 20.3979, df = 254.083, p < 2.2 × 10^-16^) as well as higher array intensities (t = 12.7456, df = 237.751, p < 2.2 × 10^-16^), indicating that differential expression of genes with high expression is detected more frequently by sequencing than by arrays.

### Modeling Sampling Error for Low Abundance Transcripts

We modeled the effect of counting noise on sequencing-based measurements of differential expression (see Methods). The scatter plots comparing differential gene expression determined from arrays and from simulated sequencing results predicted from our noise model were similar to what was experimentally observed (Fig. 1B and Fig. 1D). Both the observed results and simulations show similar increases in correlation when thresholding on the number of reads, indicating that modeling counting error captures most of the noise in the cDNA sequencing data ([see Additional file 1, Table S4] and Fig. 1E). In addition to this comparison between the RMe and RMg samples discussed above, we computed all between-strain-within-condition, and between-condition-within-strain Pearson correlations for the BYg, RMg, RMe, and RMg samples [see Additional file 1, Table S4]. The correlations showed a similar dependence on a threshold on the number of reads obtained from a gene.

### Investigating Differences by qPCR

**Figure 2:**
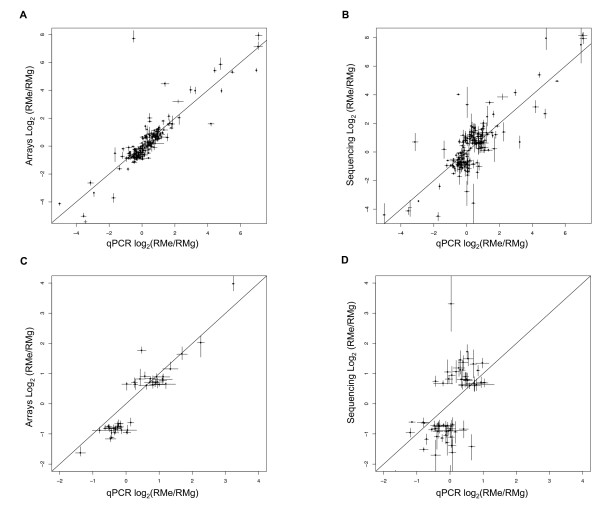
**Quantitative PCR of significantly differentially expressed genes show better agreement with arrays than sequencing**. 192 randomly sampled significantly differently expressed genes were analyzed by qPCR. qPCR results are highly correlated with both microarrays (R = 0.86, bootstrap 95% CI: 0.7043 – 0.953) (A) and sequencing results (R = .82, bootstrap 95% CI: 0.7031 – 0.8917) (B). However, the subset of the tested genes that were called significantly differentially expressed by the arrays only (see Fig. 1A, red dots) were more highly correlated (R = 0.925, bootstrap 95% CI: 0.8621 – 0.9648) (C) than the subset of genes that were called significant by sequencing (see Fig. 1A, green) (R = 0.518, bootstrap 95% CI: 0.3227 – 0.7069) (D). Error bars represent 95% confidence intervals for the differential expression measurements.

To better understand the disagreement between the cDNA sequencing and array results, we performed qPCR on a large subset of genes. We randomly selected 192 genes from genes significantly differentially expressed in the following categories: in the Illumina 1 G sequencing data only, in microarrays only, and as measured by both technologies. Of the 192 candidates, 12 genes that showed non-specific amplification or unexpected size amplification products were removed from the analysis. qPCR results are highly correlated with both microarrays (Fig. 2A R = 0.86, bootstrap 95% CI: 0.7043 – 0.953) and sequencing results (Fig. 2B R = 0.82, bootstrap 95% CI: 0.7031 – 0.8917). Of the 9 genes that were significantly differentially expressed in both technologies but where the technologies disagreed on the direction of the change, 6 out of 9 showed no differential expression by qPCR. Because of the number of genes we measured, we were able to examine the discrepant subsets more closely than previous studies[1,2]. Specifically, we found that the subset of genes recognized as significantly differentially expressed by arrays only was highly correlated with qPCR (Fig. 2C R = 0.925, bootstrap 95% CI: 0.8621 – 0.9648) whereas as the subset of genes recognized as differentially expressed only by Illumina sequencing was moderately correlated with qPCR measurements (Fig. 2D R = 0.518, bootstrap 95% CI: 0.3227 – 0.7069).

### Simultaneous Discovery of Heterozygosity and Allele-Specific Expression

As a prelude to detecting heterozygosity and measuring allele specific expression (ASE), we examined the capability of the Illumina 1 G sequencer to locate cDNA single nucleotide polymorphisms (SNPs) in our haploid RM11-1a strain samples. Alignments of ORFs from the reference sequence with the RM11-1a unfinished genome sequence provided the known position of 24,751 SNPs. We tested how accurately these known RM11-1a SNPs were rediscovered using the Illumina 1 G reads aligned with the divergent S288C reference sequence. We tested a total of 5,926,474 bases covered by at least one read. 180,628 bases showed at least one discrepancy between an Illumina base-call and the reference sequence. Considering all discrepancies gives more than a sevenfold overestimate of the known SNPs, so we calculated a likelihood ratio statistic for each discrepant base to more accurately identify SNPs (see methods). With this additional filter we were able to detect 11,608 known SNPs while only falsely calling SNPs for 457 bases (3.8 × 10^-2 ^false discovery rate).

Next, we focused on detecting heterozygosity and quantifying ASE simultaneously in data from diploid organisms. First, we simulated the results that could be expected from a diploid strain by combining the haploid BYg and RMg data sets together. This *in silico *diploid had twice the total number of reads that we obtained from a single lane for an actual diploid. Thus, it has greater power to detect sites of heterozygosity and sets an upper limit to what we could expect from an actual diploid strain. We tested a total of 6,681,784 bases covered by at least one read. 236,975 bases showed at least one discrepancy between an Illumina base-call and the reference sequence. After applying the same statistical filter described above, we were able to detect 9,848 known sites of heterozygosity while falsely calling 654 bases (6.2 × 10^-2 ^false discovery rate) for this *in silico *data set. We calculated simulated ASE expression by separating reads directly aligning over the SNP base into two groups based on which base they contain, counting the total reads in each group, and calculating the log_2 _ratio to determine differential gene expression. ASE measurements from the *in silico *BYg/RMg diploid for genes with over 10 sequencing reads were significantly correlated (R = .69, bootstrap 95% CI: 0.6504 – 0.7322) with the differential expression values calculated by summing all reads aligning to an ORF from the haploid samples.

Last, we used sequencing data obtained from an actual BYg/RMg diploid *S. cerevisiae *strain grown in glucose to discover sites of heterozygosity and simultaneously quantify allele specific expression. We tested 5,072,257 bases covered by at least one read, and 270,898 bases showed at least one discrepancy with the reference sequence. Using the likelihood ratio statistic, we were able to detect 3,691 known sites of heterozygosity while falsely calling 868 bases (1.9 × 10^-1 ^false discovery rate). We calculated ASE at these heterozygous sites by calculating the ratio of the number of reads containing the BY base and the number of reads containing the RM base. Considering only the heterozygous sites assessed by at least 10 sequencing reads, we observed limited correlation (n = 22, R = 0.313, p < 0.155) with the ASE previously quantified for those transcripts in a BY/RM diploid by allele-specific RT-PCR [14]. Although the allele-specific RT-PCR results for the BY and RM strains were obtained in a separate experiment, the RNA samples were prepared from cells grown under similar conditions.

As another assessment of ASE, we compared our ASE predictions to results from genetic linkage analysis of gene expression levels. Using 112 haploid segregants from a cross of the BY and RM strains, expression levels were linked to both local and distant loci [14]. Local linkages can be mechanistically explained by polymorphisms in promoters, 3' untranslated regions, or coding sequence [15]. Approximately 25% of all transcripts show local linkage. In many cases, these types of local linkages alter gene expression in *cis*, such that local-linkage effect sizes in a sample of transcripts have been observed to correlate with ASE as quantified by allele-specific qPCR [14]. Therefore, we compared our ASE estimates to genes with known local linkages and found that ASE ratios determined by cDNA sequencing data from the BYg/RMg diploid were weakly correlated with average effect sizes of genes with local linkages (R = 0.13, bootstrap 95% CI: 0.08 – 0.22). Thresholding on only those genes with at least 10 sequencing reads at the site of heterozygosity improves this correlation (R = 0.332, bootstrap 95% CI: 0.20 – 0.46).

## Discussion

Quantitative measurement of RNA-cDNA hybridization kinetics demonstrates that the eukaryotic transcriptome is dominated by a relatively small number of highly expressed genes[16]. 20% of the yeast transcriptome is expressed at hundreds of copies per cell, while most other transcripts are present in tens of copies per cell or fewer [17]. These predictions from hybridization data are consistent with SAGE experiments in yeast [18]. Full characterization of gene expression by sequencing requires sufficient data to accurately measure transcripts with low abundance.

We found that a large fraction of Illumina 1 G reads originated from highly abundant ribosomal rRNAs. Between 30.9%–60.4% of the total number of reads in our six samples originated from rRNAs. Even though we depleted each cDNA sample of ribosomal RNA before priming cDNA synthesis using the mRNA polyA tail, the remaining highly structured ribosomal RNAs self-primed a significant amount of reverse transcription, so that the final sample contained a large amount of cDNA originating from ribosomal RNA. This problem of limited mRNA coverage can be addressed in one of three ways: by combining data from several lanes of the sequencer (at increased cost), by developing new techniques in order to selectively and efficiently deplete abundant rRNAs, and through advances in sequencing technology that increase the number of reads per run.

The effect of the limited number of reads was observed in our model of sampling error. The most striking similarity between the simulated and real correlation plots is the vertical cluster centered at a log_2 _value of 0 (Fig. 1B and Fig. 1D). This vertical cluster suggests that small gene expression ratios were less reliably measured by the Illumina 1 G than by the Agilent microarray platform. The majority of the genes in this vertical cluster were low-abundance transcripts with few reads. Reflecting the low reliably of these measurements, filtering genes based on a threshold number of reads in all six samples improves the correlation between array and sequencing data (Fig. 1E).

The correlation between array measurements and RNA sequencing has been addressed by a number of recent studies and in most cases ranges from 0.6 to 0.85[1,2]. Similar correlations were observed when comparing microarrays to massively parallel signature sequencing (MPSS) and SAGE [19-25]. This suggests that the disparity may arise through differences between the array and sequence technologies rather than noise specific to new sequencing technologies. While correlation is a useful comparison method, a key metric for comparing expression technologies is the accuracy of detecting differential gene expression.

Counting error limits the ability of cDNA sequencing to accurately identify changes in mRNA levels for low abundance transcripts. In our analysis of the RMg and RMe samples, only 566 genes were detected as differentially expressed by both platforms, about half of the total genes found differentially expressed by each platform individually. The subset of genes that were called significantly differentially expressed by only arrays had significantly lower number of reads than the set of all genes examined, as well as lower average array intensity. Because of the limited number of filtered reads for these genes, low abundance transcripts were less accurately measured by the Illumina 1 G. In contrast, the subset of genes that were called differentially expressed by sequencing was enriched for genes with both higher read counts and higher array intensities. This indicates that additional sequencing data would improve quantification of genes with lower expression levels. Furthermore, for highly transcribed genes having more than 300 read counts and for which differential expression was assessed by arrays, sequencing, and qPCR, qPCR ratios were more strongly correlated with sequencing results than with array results (R = 0.907 vs R = 0.850). This suggested that the Illumina 1 G was less subject to saturation effects than microarray analysis.

Based on our qPCR assessment of transcript levels for a large number of genes, we find both sequencing and array technologies have similar overall accuracy for measuring differential gene expression. One report has observed very high (up to R = 0.98) correlations between cDNA sequencing and qPCR, but selected just 34 genes spanning a 6000-fold range of expression [1]. Another study used qPCR only to address discrepancies in 11 genes identified as differentially expressed [2], observing that sequencing made 4 of 5 correct calls of differentially expressed genes while arrays correctly identified 2 of 6 differentially expressed genes. Unlike our study, these studies used either much smaller sample sizes or were biased in selecting genes to be interrogated by qPCR. Because of the large sample of genes we measured by qPCR assays, we were able to examine subsets of these genes with differential expression more closely than previous studies[1,2]. We found that the subset of genes recognized as significantly differentially expressed by arrays only was highly correlated with qPCR (R = 0.925, bootstrap 95% CI: 0.8621 – 0.9648), whereas as the subset of genes recognized as differentially expressed only by Illumina sequencing was moderately correlated with qPCR (R = .518, bootstrap 95% CI: 0.3227 – 0.7069). Although caution must be exercised in distinguishing between the effectiveness of the statistical method to evaluate differential expression and the accuracy of the technology, our results suggests that arrays may have an advantage in measuring differential gene expression for low-abundance transcripts.

For 9 genes that were significantly differentially expressed in both technologies but where the technologies disagreed on the direction of the change, the qPCR results were inconclusive. All 9 genes were measured with fewer than 300 reads. The majority, six out of 9, showed no differential expression by qPCR. They were likely false positives for both sequencing and arrays or false negatives for qPCR. Two of the qPCR assayed genes agreed with the array prediction, while the third agreed with the sequencing data. These numbers were too small to draw definitive conclusions.

Our analysis, both in our comparison to arrays and qPCR, focused on the differential gene expression within the RM11-1a strain across two environmental conditions (RMg and RMe). This represents a realistic situation in which the strain of interest has several polymorphisms compared to a reference sequence. Moreover, gene expression differs greatly between these conditions [11]. The use of a non-reference strain allowed us to assess the accuracy of SNP detection in cDNA using the Illumina 1 G. Using alignments of the ORFs from a draft assembly of RM11-1a, we catalogued SNPs from genomic sequence and assessed how well these could be detected from cDNA. Simply flagging every site of sequence discrepancy from reference would have generated 180,628 predicted SNPs. The majority of these discrepancies most likely represent Illumina 1 G sequencing errors, as the sequence of RM11-1A has been established with 10× coverage Sanger sequencing [13]. Calculating a likelihood ratio statistic allowed SNP detection with high sensitivity and specificity. 11,608 known SNPs were detected, and the false discovery rate was 3.8 × 10^-2^. cDNA sequencing shows great promise for generating a coding SNP map in any genome for which a reference sequence is available.

In our assessment of heterozygous site detection in a diploid, the total number of reads available limited predictive power. In the *in silico *data set, generated by combining reads from RMg and BYg, we are able to detect 9,848 known sites of heterozygosity while falsely calling 654 sites (6.2 × 10^-2 ^false discovery rate). This performance was notably worse than detecting SNPs from haploid cDNA. Heterozygous site detection requires more reads than homozygous SNP detection because bases from each allele must be present to provide sufficient evidence for the existence of the site. This is further complicated by the presence of strong allele specific expression (ASE) for many genes, resulting in fewer reads from the less expressed allele. Consequently, our results with an actual BY4716/RM11-1a heterozygous diploid indicated that at high specificity only a smaller percentage, approximately 15%, of heterozygous sites could be detected. The discrepancy between the ability to detect heterozygosity for the *in silico *diploid and the actual BYg/RMg diploid can also be partially attributed to the fact that the *in silico *diploid was modeled by combining data from two sequencing runs and so had twice the data generated for the actual diploid.

Similarly, in our assessment of the quantitative measurement of ASE in a diploid, the total number of reads available limited the accuracy of the results. Correlation of ASE as determined by sequencing with ASE as measured by RT-PCR was weak (n = 22, R = 0.313, p < 0.1551). In contrast, ASE calculated for the *in silico *diploid showed significantly higher correlation with gene expression (R = .69, bootstrap 95% CI: 0.6504 – 0.7322). Because the *in silico *diploid contained the number of reads obtained from two lanes of a sequencer, this result suggests that the total number of reads was the limiting factor.

## Conclusion

Our analysis of next generation sequencing was motivated by the rapidly converging cost of microarray analysis with a single lane of data from an Illumina 1 G sequencer. In summary, our results indicate that, at least for the present, microarrays remain a highly competitive technology for quantitative measurement of differential gene expression. Arrays have an advantage in measuring low-abundance transcripts. Also, the current throughput of array experiments is higher than the 8 lanes present on an Illumina 1 G instrument. It is routine to process 24–96 arrays in a single day, while the Illumina instrument is limited to 8 lanes that require approximately 48 hours to run. Multiplexing approaches can increase the number of samples per lane of the instrument, but our analysis suggests that accurate analysis of gene expression in yeast by full-length cDNA sequencing requires at least the entire output of a single lane. More lanes may be necessary in other species with more complex mRNA pools.

## Methods

### Cloning of cDNAs

For BY9e, BY9g, RM9e, and RM9g, samples were identical to those described in [11]. RM-11a/BY hybrids were grown as described in [14] and total RNA was prepared by acid phenol extraction and further purified by RNEasy (Qiagen). RNEasy purification removes most RNAs smaller than 200 nt. 5 ug of total RNA were used per sample. Ribosomal RNA was depleted using the Ribominus LNA kit (Invitrogen). RNA was reverse transcribed using an anchored oligo-dT primer and Superscript III (Invitrogen). Second strand synthesis was performed using DNA polymerase I supplemented with RNaseH, and E. coli ligase (Invitrogen). The cDNAs were fragmented to a 100–200 bp length using a Covaris acoustic sample disruptor. Fragmented cDNAs were end-repaired, A-tailed, and adaptor ligated according to the genomic DNA protocol supplied by Illumina. Adapter ligation is random, so cloned double-stranded cDNAs do not retain strand information. Ligated products were gel purified and amplified with 18 rounds of PCR. Products were cleaned using a MinElute column (Qiagen) and sent for sequencing on an Illumina Genome Analyzer at the Rockefeller University Genomics Resource Center. Microarrays were performed on the Agilent 44 K S. cerevisiae gene expression platform with data obtained from hybridizations of BY9e, BY9g, RM9e, and RM9g[11].

### Aligning reads in the genome

Reads were aligned to the S288C *S. cerevisiae *genome downloaded from SGD on April 1, 2008, allowing for up to 2-edits (mismatch, insertions, and deletions). DNA bases called by the Illumina IG decrease in accuracy toward the 3' end of the read, so up to two bases were trimmed from the 3' end. We used the method of Baeza-Yates and Perleberg [26] to match reads up to 2-edits, the same method used by Illumina's own aligner Eland.

#### Algorithm

1. Index the genome using a suffix array. We used the implementation of Doring et al[27].

2. Search the genome exactly for the read, If the read did not match uniquely or in multiple locations exactly, allow 1-mismatch for the read by searching for regions of the genome that match either half of the read. Once such a region is found, use the Myers bitvector algorithm [28] to quickly confirm the rest of the read matches the location with one edit. If this read also did not match to the genome with 1-edit, allow 2-edits by searching the genome for regions of the genome that match either third of the read. Confirm these regions using the Myers bitvector algorithm.

3. For all matches within 2-edits, use the Smith-Waterman algorithm to recover an alignment.

4. For reads that did not match up to two edits, trim one base at a time from the 3' end, up to two bases, and align again using the same strategy allowing for up to 0, 1, and 2-edits.

### Computing Expression

#### Algorithm

1. Index the intervals covered by the reads in a balanced dynamic priority search tree [29].

2. Use stabbing and interval queries to obtain reads that overlap a gene's open reading frame (ORF).

3. Compute expression value for that gene as the number of reads returned by the stabbing and interval queries by gene length. If a read matched multiple locations within a single ORF, it contributed only one count for the read.

### Statistically Significant Differential Gene Expression

The following quality criteria were applied to genes in the array data: intensities well above background, no non-uniformity outliers, and no missing data. A single quality criterion was used for the sequencing data: the ORFs of genes the genes do not overlap. Statistical difference in expression for cDNA sequencing data was evaluated using Fisher's exact test on a 2 × 2 contingency table on a per gene basis in which the two columns of the table were separated by reads that overlap the gene's ORF and reads that do not overlap the ORF and the two rows were separated by the two compared samples RMg and RMe. Fisher's exact test on the contingency table corrected for overall differences in the number of filtered reads obtained for each sample. For the microarray data, statistical significance was evaluated using the Mann-Whitney test on n = 6 biological replicates; the sequenced cDNA originated from one of these 6 samples. We further corrected for multiple testing using a q-value approach. Significant difference was determined using Fisher's exact test for the cDNA sequencing data, and the Mann-Whitney test for the array data with the additional criteria of a FDR less than 1% and a 1.5 fold or greater change between the two conditions. All correlation confidence intervals reported were calculated in R using 10,000 re-samplings.

### Modeling Sampling Error for Low Abundance Transcripts

We began with the ratio of median RMe and median RMg values measured in the array experiment. We randomly perturbed the log ratio of the array data with Gaussian noise with a variance of 0.5 to model unknown sources of variance for each gene. Next, we calculated the sum of the read counts from RMe and RMg from the observed Illumina data for each gene. For each gene read counts were redistributed between the strains such that the ratio of the read counts equaled the noise-perturbed ratio from the array data. The sum of the read counts for each gene remained unchanged. For each gene a probability between 0 and 1 was generated by dividing this simulated read count by the total read counts across all genes for that strain. For each strain and each gene we simulated sequencing data by randomly sampling once from a binomial distribution defined by this probability and the total counts for each strain.

### Quantitative PCR

192 genes were selected by pooling a list of genes corresponding to those differentially expressed by arrays only, sequencing only, or both and randomly sampling once from this list. In addition, we included all 9 genes that were significantly differentially expressed in both technologies but where the technologies disagreed on the direction of the change. Primers for quantitative PCR were designed using BatchPrimer3 1.0 [30], an implementation of Primer3. Primers were selected with closely matched Tm in the range of 57 to 59 degrees surrounding a 100 bp amplicon. Primer sequences are available on request. mRNA samples were treated with RNAse-free DNAse (Invitrogen), extracted with acid phenol, and ethanol precipitated. Reverse transcription was with Superscript II (Invitrogen) using an anchored oligo dT primer. The samples were amplified in triplicate by qPCR using the Sybr Green Power PCR master mix (Applied Biosystems) on an ABI 7900 HT system. Expression levels were determined by comparison with a reference curve of yeast diploid RNA performed on the same plate.

### cDNA SNPs, Heterozygous Sites, ASE

We identified SNPs for the haploid strains and heterozygous sites for the diploid strains by calculating a likelihood ratio statistic. The likelihood that the reads for a base comes from a binomial distribution with a 2% or smaller error in sequencing any base was compared to the likelihood that comes from a binomial distribution where the probability that reads differ from the reference base is greater than the sequencing error rate. The choice of the error rate is a conservative estimate. A LOD score for all bases with at least at least one discrepancy between an Illumina base-call and the reference sequence was determined as follows:

n = total number of reads aligning to a base

y = total number of reads aligning to a base that match the reference

p = probability that reads match the reference base





Bases with LOD>3 were called SNPs or sites of heterozygosity.

## Authors' contributions

JSB and ZK analyzed the data and drafted the manuscript. AAC performed the experiments and drafted the manuscript. MS, LK designed the study and helped draft the manuscript.

## Supplementary Material

Additional File 1 **Table S1 – Read Utilization. **Table S2 – Unique Read Origin.Table S3 – Multiple Read Origin. Table S4 – Correlations of Differential Gene Expression Between Illumina 1 G and Agilent Arrays. Tables of Illumina sequencing read utilization and read origin. Correlations of differential gene expression, with and without thresholding on read coverage. Click here for file
