# Supplementary material for: Measuring differential gene expression by short read sequencing: quantitative comparison to 2-channel gene expression microarrays
Source: BMC Genomics. 2009 May 12;10:221. doi: 10.1186/1471-2164-10-221 (PMC2686739; doi:10.1186/1471-2164-10-221)
Supplement: Additional File 1 — Table S1 – Read Utilization. Table S2 – Unique Read Origin.Table S3 – Multiple Read Origin. Table S4 – Correlations of Differential Gene Expression Between Illumina 1 G and Agilent Arrays. Tables of Illumina sequencing read utilization and read origin. Correlations of differential gene expression, with and without thresholding on read coverage. [file 1471-2164-10-221-S1.doc]

Table S1 - Read Utilization

|  | **Sample Identifier** | | | | |
| --- | --- | --- | --- | --- | --- |
| **Read Utilization** | **BYg9** | **RMg9** | **BYe9** | **RMe9** | **BYg/RMg Diploid** |
| Reads with missing (N) bases | 3,338 | 6,317 | 4,745 | 4,933 | 4,795 |
| Read alignments attempted | 5,021,017 | 5,248,299 | 5,220,586 | 5,066,483 | 5,025,084 |
| Exactly, unique | 947,899 | 2,203,744 | 1,891,059 | 1,512,568 | 1,893,893 |
| Exactly, multiple | 3,225,177 | 1,908,838 | 2,411,261 | 2,361,223 | 1,398,501 |
| 1-edit, unique | 102,206 | 401,218 | 190,170 | 301,082 | 536,678 |
| 1-edit, multiple | 387,366 | 266,251 | 317,429 | 359,930 | 362,397 |
| 2-edit, unique | 38,773 | 114,865 | 61,774 | 106,037 | 241,924 |
| 2-edit, multiple | 125,262 | 88,316 | 61,774 | 144,083 | 165,068 |
| Total Aligned | **5,001,584** | **4,983,232** | **4,981,328** | **4,784,923** | **4,598,462** |
| *remaining, not aligned* | *194,333* | *265,067* | *239,258* | *281,560* | *426,622* |
| read utilization | **96.20%** | **95.00%** | **95.50%** | **94.50%** | **91.60%** |

Table S2 – Unique Read Origin

|  | **Sample Identifier** | | | | |
| --- | --- | --- | --- | --- | --- |
|  | **BYg9** | **RMg9** | **BYe9** | **RMe9** | **BRG1** |
| **ORF** | 826795 | 1996871 | 1310317 | 1165513 | 1593471 |
| rRNA | 117107 | 401286 | 560676 | 508151 | 688692 |
| tRNA | 1426 | 3995 | 5822 | 12723 | 15163 |
| Remaining | 143551 | 317675 | 266188 | 233300 | 375179 |
| Total | 1088879 | 2719827 | 2143003 | 1919687 | 2672496 |

Table S3 – Multiple Read Origin

|  | **Sample Identifier** | | | | |
| --- | --- | --- | --- | --- | --- |
|  | **BYg9** | **RMg9** | **BYe9** | **RMe9** | **BRG1** |
| tRNA only | 298 | 1205 | 455 | 321 | 499 |
| rRNA only | 3021143 | 1731071 | 2259112 | 2297356 | 1467050 |
| **one ORF only** | 41939 | 66606 | 42441 | 57598 | 77453 |
| multiple ORFs | 129503 | 165406 | 118299 | 93203 | 152139 |
| rRNA and ORF | 535298 | 290035 | 406097 | 404526 | 213572 |
| tRNA and ORF | 1907 | 3472 | 2290 | 1191 | 2064 |
| remaining | 7717 | 5610 | 9631 | 11041 | 13189 |
| Total Multiply Mapping Reads | 3737805 | 2262200 | 2838325 | 2865236 | 1925966 |

**Table S4 - Correlations of Differential Gene Expression Between Illumina 1G and Agilent Arrays**

|  | No thresholding on read counts | Thresholding on genes with >300 read counts |
| --- | --- | --- |
| **BYg vs Bye** | 0.687972 | 0.884967 (811) |
| **RMe vs Bye** | 0.49116 | 0.70658 (872) |
| **RMg vs BYg** | 0.461681 | 0.728894 (1147) |
| **RMg vs RMe** | 0.75356 | 0.90949 (1203) |

Correlations of differential gene expression. Numbers in parenthesis indicate the number of genes that pass the read count threshold.
